# Peripheral Extracellular Vesicles for Diagnosis and Prognosis of Resectable Lung Cancer: The LUCEx Study Protocol

**DOI:** 10.3390/jcm14020411

**Published:** 2025-01-10

**Authors:** Jorge Rodríguez-Sanz, Nadia Muñoz-González, José Pablo Cubero, Pablo Ordoñez, Victoria Gil, Raquel Langarita, Myriam Ruiz, Marta Forner, Marta Marín-Oto, Elisabet Vera, Pedro Baptista, Francesca Polverino, Juan Antonio Domingo, Javier García-Tirado, José María Marin, David Sanz-Rubio

**Affiliations:** 1 Pulmonology and Critical Care Unit, Hospital Universitario Miguel Servet, 50009 Zaragoza, Spain; 2 Translational Research Unit, Hospital Universitario Miguel Servet, IIS Aragón, 50009 Zaragoza, Spain; 3 CIBER Enfermedades Respiratorias, 28029 Madrid, Spain; 4 Thoracic Surgery Unit, Hospital Universitario Miguel Servet, 50009 Zaragoza, Spain; 5 Instituto de Investigación Sanitaria de Aragón (IIS Aragón), 50009 Zaragoza, Spain; 6 Division of Pulmonary and Critical Care Medicine, Department of Internal Medicine, Baylor College of Medicine, Houston, TX 77030, USA

**Keywords:** lung cancer, diagnosis, prognosis, extracellular vesicle, exosomes, micro-RNA

## Abstract

**Background/Objectives:** Lung cancer is the primary cause of cancer-related deaths. Most patients are typically diagnosed at advanced stages. Low-dose computed tomography (LDCT) has been proven to reduce lung cancer mortality, but screening programs using LDCT are associated with a high number of false positives and unnecessary thoracotomies. It is therefore imperative that a certain diagnosis is refined, especially in cases of solitary pulmonary nodules that are difficult to technically access for an accurate preoperative diagnosis. Extracellular vesicles (EVs) involved in intercellular communication may be an innovative biomarker for diagnosis and therapeutic strategies in lung cancer, regarding their ability to carry tumor-specific cargo. The aim of the LUCEx study is to determine if extracellular vesicle cargoes from both lung tissue and blood could provide complementary information to screen lung cancer patients and enable personalized follow-up after the surgery. **Methods:** The LUCEx study is a prospective study aiming to recruit 600 patients with lung cancer and 50 control subjects (false positives) undergoing surgery after diagnostic imaging for suspected pulmonary nodules using computed tomography (CT) scans. These patients will undergo curative surgery at the Department of Thoracic Surgery of the Miguel Servet Hospital in Zaragoza, Spain, and will be followed-up for at least 5 years. At baseline, samples from both tumor distal lung tissue and preoperative peripheral blood will be collected and processed to compare the quantity and content of EVs, particularly their micro-RNA (miRNA) cargo. At the third and fifth years of follow-up, CT scans, functional respiratory tests, and blood extractions will be performed. **Discussion:** Extracellular vesicles and their miRNA have emerged as promising tools for the diagnosis and prognosis of several diseases, including cancer. The LUCEx study, based on an observational clinical cohort, aims to understand the role of these vesicles and their translational potential as complementary tools for imaging diagnosis and prognosis.

## 1. Background

Lung cancer has the highest mortality rate among all cancer types. Its risk factors are widespread and its subtle symptoms often lead to delayed diagnosis until the disease has significantly progressed. In 2022, it accounted for a total of 2,480,301 cases worldwide, representing 12% of all cancers, with a total of 1,817,172 deaths, making up 18.7% of all cancer-related deaths [1].

The clinical manifestation of lung cancer is often nonspecific and frequently presents with few symptoms, which may correlate with the disease’s extent. Up to 25% of patients are asymptomatic at the time of diagnosis [2]. Around a third of affected patients typically experience dyspnea, while other presentations may include chest pain, hemoptysis, pulmonary embolism, pneumothorax, and pleural or pericardial effusion [3]. Various chest structures, such as lymph nodes, may suggest invasion and lead to secondary symptoms [2]. Conversely, distant metastases can cause a wide range of symptoms [2].

Despite advancements in treatment and understanding molecular targets, along with broader therapeutic options, the survival rates and prognosis for lung cancer patients remain poor [4,5]. While maintaining a commitment to treatment and the focus on tumor cells, it is imperative to explore the early stages and strive for improved early diagnosis, with the clear goal of preventing the loss of years and quality of life for our patients. Screening programs have been developed, showing a 19% reduction in mortality in the largest case series. However, these programs have not yet been widely implemented and have presented around a 3% rate of false positives [6,7].

The subtle symptoms often lead to delayed suspicion. In daily clinical practice, chest radiographs are commonly performed in various scenarios. However, Bradley et al. conducted a systematic review on the sensitivity of this test and estimated that in patients already presenting symptoms, it failed to detect cancer in up to 20% of cases. [8]. Accumulated evidence supports the use of imaging tests, particularly LDCT, in heavy smokers, significantly reducing mortality and enabling early detection of lung cancer [6]. Since the publication of the National Lung Screening Trial results in the United States, this type of screening has become the standard of care. Nevertheless, implementation in Europe has been slow, partly due to the need for more solid evidence [6,9].

Lung tumors are classified into stages according to the eighth edition of the TNM classification, approved in January 2017 [10]. The optimal treatment in stages I and II is surgery if there are no formal contraindications, as they are potentially curable. Stage III is a heterogeneous group that includes resectable lesions, potentially resectable lesions, and locally advanced but inoperable lesions, which require evaluation by an expert committee. Stage IV encompasses lesions with metastatic spread [11], not fit for resection. The World Health Organization (WHO) also proposes a histological classification of lung lesions. Up to 18 types of lung tumors are included, with the most relevant ones being adenocarcinoma, squamous cell carcinoma, neuroendocrine tumors (including small cell carcinoma), large cell carcinomas, mesenchymal tumors, and metastases from other primary tumors [12,13].

New treatments have increased life expectancy, giving rise to the increase in long-term survivors, defined as those who survive for more than 5 years from diagnosis. In the statistical measurement of cancer patient survival, traditionally, the 5-year survival rate following diagnosis is considered a measure of medium-term mortality. However, the intrinsic characteristics of lung cancer and clinical experience have shown that this measurement is imprecise, as recurrence can occur much later, requiring close monitoring [11,14,15]. This circumstance, coupled with the significant cost of the disease and the loss of years of life, underscores the need to seek new tools beyond clinical monitoring and successive CT scans [16] that allow us to anticipate recurrences.

It is crucial to highlight the issue of overdiagnosis, which involves identifying tumor lesions that wouldn’t significantly affect the patient’s lifespan or cause symptoms before their death. Quantifying this phenomenon is complex, but it has been reported to exceed 60% in some series [6]. There is a need, therefore, for a non-imaging-based biomarker that enables the clinician and the patient to determine whether surgery is necessary.

## 2. New Perspectives: Extracellular Vesicles

Recently, the concept of “liquid biopsy” has emerged as a potential diagnostic tool. This concept involves easily accessible body fluids such as blood, urine, serous effusions, or saliva to overcome the limitations of traditional biopsy [5]. Extracellular vesicles (EVs) are heterogeneous cellular vesicular structures surrounded by a lipid bilayer that play a role in cell-to-cell communication. Among them, exosomes, microvesicles, and apoptotic bodies can be distinguished. They are released by most cell types and have been described in several body fluids, including plasma and serum [17,18].

Exosomes, ranging from 40 to 150 nm in diameter, are formed by the invagination of the cell membrane and fuse with intracellular vesicles. The vesicular content is diverse and may include nucleic acids, proteins, lipids, amino acids, and metabolites [18]. Vesicular transport is selectively regulated and is altered in tumor cells, leading to vesicular contents different from physiological ones, which can consequently alter the functions of the cells they encounter [5]. This circumstance can be studied and potentially serve as a diagnostic and therapeutic marker [19,20,21,22,23,24].

## 3. Materials and Methods

### 3.1. Study Design and Setting

The LUCEx study is a 5-year non-interventional, longitudinal, prospective study being conducted at the Miguel Servet University Hospital, a major teaching hospital in Zaragoza, Spain. Before the surgical procedure for the removal of suspicious lesions identified via CT scan, clinical data and peripheral blood will be collected. Then, intra-operatory normal lung tissue will be collected as well. Three years following the surgery, new blood extraction and pulmonary function tests (PFTs) will be conducted to evaluate analytical and functional variations in patients. All measurements will be repeated 5 years after the surgery. All participants will receive standard follow-up care from their family doctor, as well as from their pulmonologist, thoracic surgeon, or oncologist if required, according to international guidelines, with no additional cost to the patient.

### 3.2. Study Objectives

1. Establish a cohort to determine the incidence of different cancer subtypes in patients with surgically resectable lesions and identify the rate of false-positive findings on CT scans suggestive of malignant lesions to assess its sensitivity.
2. Evaluate and quantify the presence of exosomes in the blood plasma of patients with and without cancer, studying their content both qualitatively and quantitatively to find out if there are statistical significant differences.
3. Investigate the differential expression of exosomal contents, if any, according to the pathology of the patients from whom they originate to detect if exosomes are a useful tool as a form of diagnosis.
4. Determine new diagnostic biomarkers based on the study of exosomes in liquid biopsy. Evaluate the behavior of these markers depending on the clinical evolution during follow-up.
5. Study of extracellular vesicles not only in liquid, but also in solid tissue to determine prognostic biomarkers to assess the differences and the systemic impact of local disease.
6. Conduct a physiopathological study of the tumor microenvironment both locally and distantly based on the study of extracellular vesicles.

### 3.3. Participants

The patients enrolled in this study will be selected by the Regional Department of Thoracic Surgery of Aragon (Spain), which includes the Miguel Servet University Hospital and the Lozano Blesa University Clinical Hospital, before their surgical intervention.

We will recruit patients during their diagnostic study for radiological images obtained through CT scans suggestive of malignancy and amenable to surgical intervention with curative intent. The diagnostic study will be conducted collaboratively by the Pneumology and Radiology Units of various hospitals within the Aragonese Health Service, with the collaboration of affiliated Health Centers. Following the appropriate assessment of surgery indication and feasibility, all patients will undergo surgery at the Thoracic Surgery Unit of Miguel Servet University Hospital and will be histopathologically studied by the Pathology Service of the same hospital. Patients will be informed prior to their inclusion in the LUCEx study and will provide their acceptance by signing an informed consent form. The Institutional Review Board of the Aragón Institute of Health approved the LUCEx study (C.I. PI24/438; 6 November 2024, Acta 20/2024).

It is not expected that all lesions will be cancerous or malignant, allowing us to establish two study groups within our sample: patients with cancer and patients without cancer. The inclusion and exclusion criteria for both groups are shown in Table 1.

After surgery and complete resection with margins, a piece of healthy lung parenchyma measuring approximately 2 cm^2^, away from the tumor area but from the same lobe, will be provided for study. Healthy lung tissue comes from surgical margins that are usually respected and does not imply an additional loss of parenchyma for the patient.

Additionally, peripheral blood will be extracted from the same patients via venipuncture before surgery and anesthesia.

### 3.4. Measurements

#### 3.4.1. Clinical Data

From each patient, basic data such as age, sex, race, and occupation will be collected, along with anthropometric measurements (height, weight, and BMI). Regarding clinical data, we will gather information on tobacco and alcohol exposure, as well as the presence of hypertension, diabetes, dyslipidemia, and any previous diagnosis of asthma or COPD. PFTs with DLCO (diffusing capacity of the lungs for carbon monoxide) will be performed according with current American Thoracic Society/European Respiratory Society guidelines [25]. CT images will be reviewed for findings such as emphysema, bronchiectasis, coronary calcium, or osteoporosis. The type of surgery and the length of hospital stay will be noted. Finally, the histological tumor diagnosis with its individual characteristics will be recorded and used to sort patients by type of cancer. We will periodically review the database to monitor cases of recurrence or any changes that occur.

#### 3.4.2. Primary Sample Processing

Once the tissue and blood samples intended for study are obtained, they will be subjected to the established protocol for cryopreservation and subsequent analysis.

##### Lung Tissue

The lung tissue fragment received from the operating room will be processed immediately after surgery, following strict sterile conditions within a hood. A macroscopic image of the fragment will be captured before it is divided into smaller pieces. These pieces will then be divided into two 5 mL microcentrifuge tubes. Subsequently, the tubes will be rapidly placed in a freezer at −80 °C to preserve them until the time of analysis.

##### Peripheral Blood

Similarly to the lung tissue sample, the peripheral blood sample will be processed immediately before surgery. The sample will be collected in a vacuum tube with EDTA. To separate the different blood fractions, the sample will be centrifuged for 15 min at 3000 rpm (revolutions per minute). After centrifugation, the plasma sample will be aliquoted first, followed by the white cell ring, and finally the red blood cells will be collected. All aliquots will be frozen at −80 °C, awaiting analysis.

##### Isolation of Exosomes

In this study, we plan the isolation of EVs for both sample origins, concretely exosomes, with commercial precipitation methods. We have previously developed an expertise in these methods, including modified steps to improve the yield and purity.

Below is the detailed procedure employed for each type of sample.

##### Lung Parenchyma

**Figure 1:**
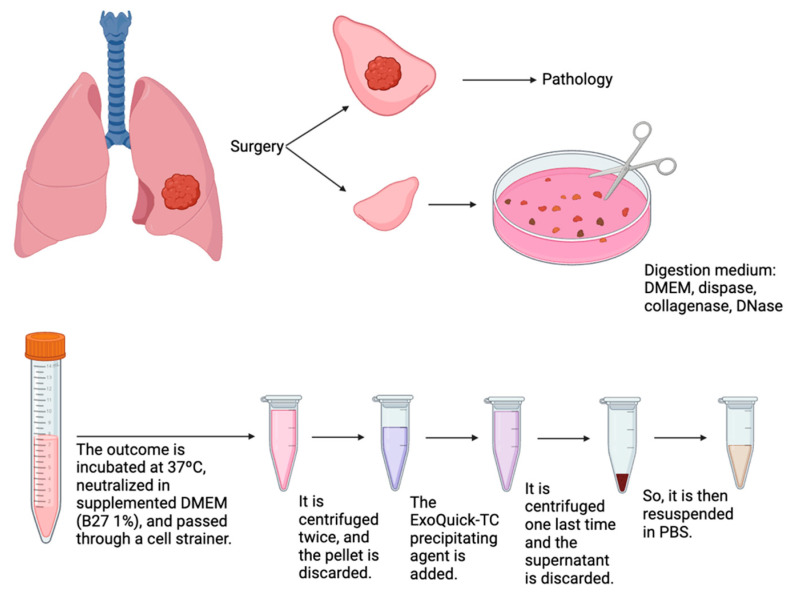
Exosome isolation process in solid tissue (created in BioRender.com).

This origin presents a particularity regarding the type of sample described in the literature and in pre-established isolation kits, so it will first be necessary to adapt the sample to a suspended state through enzymatic digestion, followed by certain adaptations of the protocol recommended by the commercial company for the application of the precipitation kit. Firstly, the tissue will be thawed and weighed to prepare the enzymes constituting the digestion medium. The digestion medium will consist of DMEM medium along with collagenase, DNase, and dispase. As shown in Figure 1, the thawed tissue will be placed in a dish and cut with scissors immersed in the medium, aiming to expose the maximum surface area to the solution. Subsequently, it will be incubated at 37 °C for two hours with agitation. Once this process is completed, the reaction will be neutralized with 1% B27-supplemented DMEM, and the resulting liquid will be divided into 1.5 mL microcentrifuge tubes. The B27 supplement is a mixture of Vitamin E, Vitamin E acetate, superoxide dismutase, catalase, glutathione, and Vitamin A.

After homogenization, the isolation of exosomes will proceed. Firstly, two centrifugations will be performed. The first at 6000 rpm (2000× *g*) for 20 min at 20 °C, and the second at 12,000 rpm (10,000× *g*) for another 20 min at 20 °C. The pellet will be discarded on both occasions, and the supernatant will be retained. At this point, the ExoQuick-TC (System Bioscience, Palo Alto, CA, USA) precipitating agent will be added, and the samples will be incubated overnight at 4 °C.

After incubation, a centrifugation at 12,000 rpm (10,000× *g*) for 75 min at 4 °C will be performed, and this time the supernatant will be discarded, and the pellet will be retained, which will then be resuspended in PBS. PBS, or phosphate-buffered saline, is an isotonic and non-toxic solution for cells that, thanks to the phosphate groups it contains, can maintain a stable pH, being widely used in biomedical research. Finally, the microcentrifuge tubes will be stored at −20 °C.

##### Peripheral Plasma

The selected fraction for exosome isolation from peripheral blood is plasma. After thawing a 600 µL aliquot, two centrifugations will be performed, like those performed with lung tissue. The first centrifugation will be at 6000 rpm (2000× *g*) for 5 min at 20 °C, and the second one at 12,000 rpm (10,000× *g*) for 20 min at 20 °C, discarding the sediment or pellet each time.

**Figure 2:**
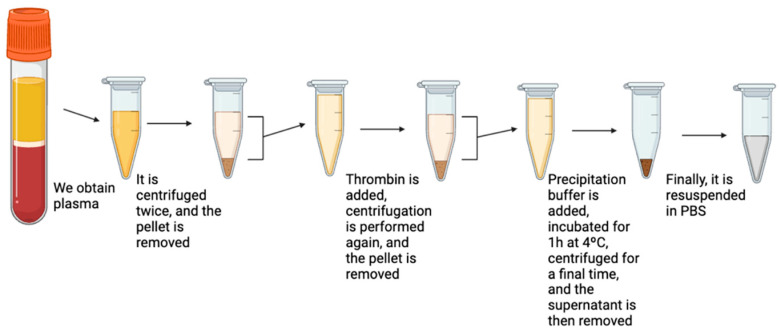
Exosome isolation process in blood plasma (created in BioRender.com).

Next, 0.6 µL of thrombin will be added, and the sample will be centrifuged again at 12,000 rpm (10,000× *g*) for 5 min at 20 °C, discarding the sediment. Then, the precipitation solution will be added, and the sample will be incubated for 1 h at 4 °C. Figure 2 schematically illustrates the different isolation steps.

Afterward, a final centrifugation will be performed, discarding the supernatant, and the sediment will be resuspended in PBS. The suspension will be stored in microcentrifuge tubes at −20 °C.

#### 3.4.3. Exosome Characterization

After isolating exosomes from our samples, and considering their microscopic nature, various techniques for characterization and quantification will be employed to confirm their presence. This will involve analyzing protein markers as well as their morphology.

##### Protein Characterization: ELISA

The presence of protein markers will be confirmed, particularly those derived from tissue, using additional techniques. Specifically, antibodies against CD63, CD81, or EPCAM will be used for detection through Western blot analysis. Furthermore, flow cytometry will be employed to detect specific markers (CD9, CD63, and CD81) using the ExoFlow 2.0 kit (System Biosciences, Palo Alto, CA, USA). The protein amount in the samples will be measured to obtain a concentration of 100 µg.

##### Morphological Characterization: Transmission Electron Microscopy

This procedure will be conducted in collaboration with the Biological Samples Microscopy Service of the University of Zaragoza, located at the Faculty of Medicine. At least five samples from each group will be prepared for obtaining representative images.

Initially, the samples will be diluted 1:50 in PBS. Subsequently, they will undergo fixation and negative staining with uranyl acetate on a layer of activated carbon. The visualization of the samples will be conducted by a specialized technician from the Institute of Nanoscience and Materials of Aragon (INMA).

##### Quantification of Exosomes: NTA

As the final technique for characterization, and aiming to quantify the exosomes in our samples, we will perform Nanoparticle Tracking Analysis (NTA). This analysis calculates the distribution of particles based on their light scattering properties and Brownian motion to determine their concentration and analyze their trajectory. The study will be conducted using a Nanosight NS300 instrument from Malvern Panalytical (Malvern, UK). The samples will be diluted 1:1000 in a solution containing PBS and EDTA to prevent aggregation. The image will be recorded by the tracking analysis software, which will then analyze and calculate their hydrodynamic diameter using the Stokes–Einstein equation [26,27].

#### 3.4.4. Small-RNA Analysis

Small-RNA analysis will focus on miRNAs and will consist of two phases.

The first phase, the exploration analysis, will be performed using the RNA sequencing (RNA-Seq) technique [28]. This method allows for the determination of the quantity, presence, and types of RNA contained within a given biological sample. The technique utilizes RNA to obtain double-stranded DNA through reverse transcription PCR. Once this DNA strand is obtained, it will be amplified to create a DNA library, which will then be fragmented into pieces ranging from 30 to 400 base pairs. These fragments will be sequenced to compare the results with a genomic or transcriptomic reference model to characterize the sample [28,29]. This technique will enable the differentiation of various RNA types, as well as specific quantitative changes in each subject. The RNA-Seq procedure will be conducted externally.

The second phase, the validation analysis, will be conducted in our laboratory, where we will evaluate the differential miRNAs found in the exploration analysis using real time quantitative PCR (RT-qPCR). For this procedure, we will utilize the LNA technology developed by Qiagen (Hilden, Germany). Each sample and miRNA will be evaluated in triplicate, and a differential expression analysis (2−∆∆Ct) will be applied.

### 3.5. Power Considerations

The sample size calculation for LUCEx is estimated to be 600 patients. Lung cancer is primarily categorized into non-small cell lung cancer (NSCLC), which constitutes 85% of patients, and small cell lung cancer, which accounts for 15% of patients [30]. We do not expect to find a similar incidence as reported for small cell lung cancer, as this subtype is rarely eligible for surgery due to its inherent conditions, and it has a much poorer survival rate compared to non-small cell lung cancer [31]. We estimated the sample size with G*Power software (version 3.1) based on our preliminary data obtained in lung adenocarcinoma patients. With these data, the effect size of our sample was 0.666. Then, we considered an α error of 0.05, a power (1 − β error) of 0.95, and an allocation ratio of 0.3. The sample size with these requirements were 39 control subjects and 129 lung adenocarcinoma patients.

**Figure 3:**
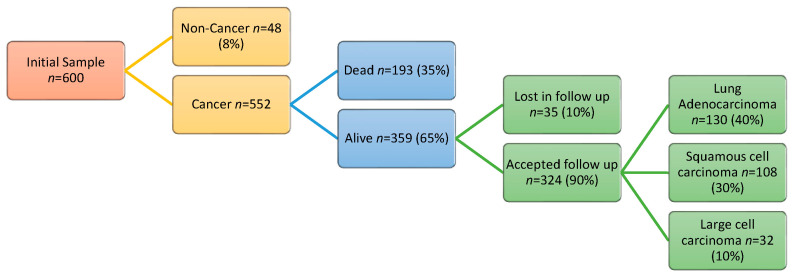
Planned flowchart for the sample size described.

Figure 3 shows the predicted flowchart to assess the sample size described. The controls will be obtained from false positives after surgical resection, in those patients whose CT findings suggest malignancy but are ultimately ruled out by histology. The percentage of controls is expected to be low, around 8% (*n* = 48).

A large sample size of lung cancer (*n* = 552) is necessary, since the survival rate for this type of cancer remains low [32]. After survival prediction, 359 patients are expected to be able to complete the follow-up. However, we also considered a loss of 10% due to the refusal to come to the follow-up visits. Among 324 non-small cell lung cancer patients expected to complete the study, according to previous reports [30], the main subtypes will be adenocarcinoma (40%, *n* = 130), squamous cell carcinoma (30%, *n* = 108), and large cell carcinoma (10%, *n* = 32). We plan to recruit at least 100 patients per year with the goal of having a preliminary study within two years of starting recruitment.

### 3.6. Statistical Analysis

In the table depicting the clinical characteristics of the population, quantitative data are shown as mean and standard deviation (SD) or median and interquartile range (IQR). These variables include age, Charlson Comorbidity Index, Body Mass Index (BMI), PFT data, or smoking history, among others. The remaining variables such as sex, smoking status, presence of hypertension, dyslipidemia, diabetes, COPD, or bronchiectasis will be presented as absolute numbers and frequencies.

Groupwise comparisons (*n* > 2) will be performed using a nonparametric Kruskal–Wallis test followed by a Mann–Whitney U test. A chi-square test will be performed for categorical variables. To compare exosome quantification between samples with cancer and controls, the Mann–Whitney test will be used, with a significance threshold of 0.05. For the RNA-Seq study, DESEq2 (R) will apply to determine differential expression of miRNAs, with a false discovery rate (FDR) of 5%. TargetScanHuman 8.0 will predict the targets of differentially expressed miRNAs, and gene ontology (GO) and Kyoto Encyclopedia of Genes and Genomes (KEGG)’s enrichment analysis will be applied. Pearson’s or Spearman’s correlation, depending on the variables’ normality, will be used to evaluate the relationship between miRNAs and clinical variables. To evaluate miRNAs in the validation analysis, the Mann–Whitney test will be used, with a significance threshold of 0.05. Additionally, ROC curve analysis and Kaplan–Meyer analysis will be carried out to evaluate the translational potential of selected miRNAs. R (R software, version 4.4.1), GraphPad Prism 9 (GraphPad Software) and SPSS version 23.0 (IBM) statistical packages will be used for the analysis.

## 4. Discussion

The Extracellular Vesicles from Lung Parenchyma (LUCEx) study focuses on patients with lung lesions suggestive of malignancy, particularly aiming to identify differential miRNA expression in patients with lung adenocarcinoma, thereby elucidating the systemic expression mechanisms of the disease. Through molecular understanding, the study seeks to achieve early diagnosis and improve clinical outcomes in terms of quality of life and survival.

Lung cancer presents a significant global health challenge. According to the most recent data provided by the World Health Organization through its observatory, in 2020, there were 19.3 million diagnosed cancer cases were worldwide, with lung cancer ranking as the second most frequently diagnosed cancer with 2.2 million affected individuals (11.4% of cases) and the leading cause of death (18%, 1.8 million individuals) [33].

Over the years, several clear risk factors have been identified in relation to the neoplastic transformation of lung tissue. It is important to note that, besides exposures, there is a clear familial aggregation in the presentation of the cases [34].

The most significant risk factor is tobacco smoke, which is associated with up to 90% of lung cancer cases [30]. The carcinogenic potential of tobacco is attributed to polycyclic aromatic hydrocarbons and nitrosamines present in the smoke. Despite increasing the risk from 10 to 30 times, and 80% of cases occurring in smokers, only 20% of smokers develop this disease [3,4].

Among chronic pulmonary pathologies, the risk of developing lung cancer is six times higher in patients with COPD and two times higher in patients with alpha-1 antitrypsin deficiency [4]. Additionally, patients with pulmonary fibrosis may also present an increased risk [35]. It is important to note alterations in normal immune function, which impair its ability to eliminate sporadically arising tumor cells. Therefore, HIV infection, alcohol abuse, or diabetes could also be considered as independent predisposing factors [35,36,37].

Considering individual risk factors, the presence of estrogen receptors in tumor lung tissue has suggested the influence of female hormones on tumor proliferation and disease prognosis. A lower risk of lung cancer has been described in women with late menarche and those who received antiestrogen therapy for breast cancer, reinforcing this theory, especially in never-smoking women [35]. Genetic polymorphisms related to the risk of disease, specifically alterations in genes that regulate cytochrome P450 enzymes, have also been identified. Two of them are found in exon 7 of CYP1A1: the T3801C (MspI) and A2455G (Ile462Val) substitutions [38]. Another polymorphism in CYP1B1, specifically the Leu432Val, also increases the risk independently of tobacco exposure [39]. Both enzymes are involved in the metabolism of polycyclic hydrocarbons and can metabolize them into intermediate carcinogens.

Apart from susceptibility, once suspicion is established, there are clear and daily difficulties in the diagnostic process. Tumor tissue is often heterogeneous, and taking a biopsy can be an incomplete method of characterizing the tumor [5,40]. It is also worth noting that obtaining a significant and adequate biopsy is not without potential complications.

During follow-up, it is known that tumor histology can change, and some authors support re-biopsy to guide evolving treatment [41]. Long-term follow-up also raises uncertainties, and discussing a disease-free cure implies de-escalating care, which can put some patients at risk, as recurrences can occur long after the initial diagnosis [15]. Hubbard et al. conducted a follow-up study on a large cohort of lung cancer patients for up to 18 years after diagnosis, revealing that disease-specific survival progressively decreases over time. In this study, among patients who survived more than 10 years free of disease, 40.6% died from causes directly related to lung cancer. Regarding overall survival, only 24.3% of patients from this sample reached the end of the study [15].

Exosomes, produced ubiquitously in the body, are a fundamental part of intercellular communication. They are regulated by the proteins contained in their membrane and are capable of re-fusing and interacting with other cells [42]. Their final content is diverse and may include nucleic acids, proteins, lipids, amino acids, and metabolites [18]. This content is selectively regulated by the ESCRT (Endosomal Sorting Complex Required for Transport), which is altered in tumor cells and can result in vesicular contents different from physiological ones, thereby altering the functions of the vesicles and consequently the cells they come into contact with [5].

Intercellular interaction through exosomes can occur via fusion, endocytosis, and receptor-mediated binding [42]. The function of the exosome, as described, depends on its cell of origin and its content. Most of them are involved in maintaining homeostasis, although they are also associated with immune response, viral pathogenesis, pregnancy, cardiovascular diseases, central nervous system diseases, and cancer progression, among others [18,43].

More specifically, and focusing on their role in lung cancer, exosomes influence neoplastic origin, tumor growth, metastasis formation, angiogenesis, immune dysregulation, creation of a suitable tumor microenvironment, paraneoplastic syndromes, and therapy resistance [18,42]. Studies such as that of Rabinowits et al. have demonstrated an increase in plasma exosome levels in patients with lung adenocarcinoma compared to healthy control groups [44]. Additionally, in another study by Rodríguez et al., it was shown that exosome levels were also significantly elevated in bronchoalveolar lavage samples from individuals with cancer compared to healthy patients [45]. Within the range in exosome contents, particular attention has been given to miRNAs. Studies have identified various miRNAs, such as miR-205-5p and miR-200b, which were statistically significant in a sample as diagnostic markers to distinguish between cancer and pneumonia [46].

The rationale for conducting this study stems from the urgency to address the challenges posed by lung cancer diagnosis and treatment. The diagnostic potential of exosomes is yet to be fully discovered, but it could be the key.

## Figures and Tables

**Table 1:** Inclusion and exclusion criteria.

Selection Criteria	
Inclusion Criteria	Exclusion Criteria
All sex/gender. Referred patient for pulmonary surgery. Signed consent and willingness to participate in follow-up.	Age over 75 years old. HIV positive. Confirmed exposure to *M. tuberculosis* without correct preventive therapy. Repeatedly positive non-tuberculous mycobacteria cultures. Pneumoconiosis (asbestosis, silicosis…). Known and unresolved hypersensitivity pneumonitis. Multiple surgical procedures involving lung or chest wall. Lung transplant patient. Metastatic, locally advanced, or with lymph node involvement lung cancer. Endobronchial brachytherapy. Pleural or chest wall cancer. Any type of cancer, up to 5 years after complete remission.

## Abbreviations

BMI	Body Mass Index
CD9	Cluster of differentiation 9
CD63	Cluster of differentiation 63
CD81	Cluster of differentiation 81
COPD	Chronic obstructive pulmonary disease
CT	Computed tomography
DLCO	Diffusing capacity of the lungs for carbon monoxide
DMEM	Dulbecco’s Modified Eagle Medium
DNA	Desoxirribonucleic acid
EDTA	Ethylenediaminetetraacetic acid
EPCAM	Epithelial cell adhesion molecule
ELISA	Enzyme-linked immunosorbent assay
ESCRT	Endosomal sorting complex required for transport
EV	Extracellular vesicles
FDR	False discovery rate
GO	Gene ontology
HIV	Human immunodeficiency virus
IQR	Interquartile range
INMA	Institute of Nanoscience and Materials of Aragon
KEGG	Kyoto Encyclopedia of Genes and Genomes
LDCT	Low-dose computed tomography
LUCEx	Lung cancer extracellular vesicles
LNA	Locked nucleid acid
miRNA	micro-RNA
NTA	Nanoparticle Tracking Analysis
NSCLC	Non-small cell lung cancer
PBS	Phosphate-buffered saline
PFTs	Pulmonary function tests
RNA	Ribonucleic acid
RNA-Seq	RNA Sequencing
RT-qPCR	Real-time quantitative polymerase chain reaction
SD	Standard deviation
SPSS	Statistical package for the social sciences
TNM	Tumor, node, and metastasis
WHO	World Health Organization
